# Novel Cr/Si-Slurry Diffusion Coatings for High Temperatures

**DOI:** 10.3390/ma16237480

**Published:** 2023-12-02

**Authors:** Michael Kerbstadt, Emma Marie Hamilton White, Mathias Christian Galetz

**Affiliations:** Dechema-Forschungsinstitut, 60486 Frankfurt am Main, Germany; michael.kerbstadt@dechema.de (M.K.); emma.white@dechema.de (E.M.H.W.)

**Keywords:** diffusion coatings, chromium slurry coating, chromium-silicon coating, high-temperature oxidation

## Abstract

Surface enrichment in Al, Si, and Cr can greatly improve high temperature oxidation resistance of many alloys. Al, Si, and Cr coatings are commonly applied via simple slurries or more complex pack cementation processes. Due to the high melting point of Cr, the deposition of Cr-based diffusion coatings by the slurry technique has proved challenging, and to date, Cr has mostly been applied by pack cementation. Here, a novel Cr-Si coating process via the slurry technique is described which has been developed and then demonstrated on two Ni-based superalloys, Rene 80 and Inconel 740H. The addition of Si to the slurry lowers the melting point via a Cr-Si eutectic and enables the formation of a liquid phase during heat treatment. Through this Cr-Si slurry coating process diffusion layers enriched by Cr and Si of about 150 µm were achieved. Oxidation behavior was studied through isothermal exposures at 900 °C for 1000 h in lab air. Uncoated Rene 80 and IN740H both showed formation of a Ti-containing Cr_2_O_3_ scale below a thin TiO_2_ top layer. Underneath the external scale a zone of internally oxidized Al grew over the exposure time and reduced the load-bearing cross-section progressively. In comparison, the Cr/Si-coated samples did not show internal Al oxidation, but a slow-growing Si-rich oxide film underneath the external Cr_2_O_3_ scale. This subscale represents an additional oxygen diffusion barrier. Thus, the weight gain during exposure for the coated samples was significantly lower than for the uncoated materials.

## 1. Introduction

Ni-based superalloys are commonly used for high-temperature environments due to their outstanding mechanical properties and high temperature strength (up to 1150 °C). To also ensure sufficient oxidation and corrosion resistance at high temperatures, diffusion coatings are widely used to enrich the surfaces in Al, Cr, and/or Si. With high Al, Cr, and/or Si concentrations protective oxide scales form on the alloys [1,2,3,4].

The state-of-the-art industrial process for chromizing is pack cementation. In pack cementation, components are usually fully embedded into a powder mixture containing the metallic source for the coating and a halogenide activator. During the heat treatment, the halogen activator transports the coating elements via the gas phase to the substrate surface, where they are deposited and afterwards diffuse into the substrate [1,5,6]. Due to the powder embedding step, the process is highly material-, energy- and labor-intensive. 

An alternative process with high economic potential is a slurry coating, where the slurry contains the metallic source (also as a powder). This liquid mixture is deposited directly on the substrate surface. Slurry deposition can occur via airbrushing, painting, or dipping. During a post heat-treatment, the diffusion coating forms by interdiffusion between the slurry particles and the substrate surface. Compared to pack cementation, the slurry process offers the potential of localized heat treatments and coating larger dimensions much more easily [7]. In order to achieve sufficient diffusion rates to form a dense layer, the existence of a liquid phase during the process is highly desirable [1]. Because of the low melting point of aluminum, the slurry process is well established for Al-based diffusion coatings and widely published in different variations [7,8,9,10,11,12]. The high melting point of pure chromium, above 1900 °C, does not allow the required heat treatment temperatures to form a liquid phase without melting, e.g., commercial Ni-base superalloys. However, Cr-based coatings can be more beneficial than Al-based coatings, e.g., at temperatures below 900 °C in sulfur-rich hot corrosion environments [13]. The deposition via the slurry technique has only been demonstrated for Cr with the use of additional halogenide activators, where the halogenides promote the generation of Cr-containing vapors from the slurry and deposition via the gas phase, basically being a pack cementation process with less powder [14,15,16]. 

Motivated by the reduction of energy and process costs, Cr/Si-based slurry coatings have recently been patented [17] and are introduced in this work. These coatings represent a new and environmentally friendly (without extra halogenide additions) approach to apply Cr/Si-based diffusion coatings on Ni-based superalloys. Due to the partially liquid state from a Cr-Si eutectic, higher Cr activities can be achieved, allowing thicker coatings after shorter heat treatment times when compared to pack cementation.

The addition of Si is beneficial as it reduces the melting temperature within the binary Cr-Si system [18]. Even better, it forms lower melting eutectics in the ternary system, e.g., Lugschneider et al. [19] demonstrated a ternary eutectic at 1077 °C for the composition of Ni-20at.-%Cr-21.1at.-%Si, according to the phase diagram, the lowest occurs at 966 °C [20]. Thus, the formation of Cr-containing liquid phases becomes possible at temperatures below 1200 °C either by interdiffusion with the Ni-based substrate or through direct addition of Ni to the slurry [21,22]. Beyond lowering the melting temperature, Si has the additional advantage of providing beneficial effects in high temperature oxidative and corrosive environments. In the past, Cr/Si-diffusion coatings have been accomplished by a co-deposition single step pack cementation process, where Si was found to be beneficial for the oxidation behavior and attributed to a slow growing SiO_2_-subscale underneath the outer Cr_2_O_3_-scale [23,24]. This Si-rich oxide subscale acts as an additional diffusion barrier to oxygen uptake into the alloy [25]. It is usually amorphous and crystalized SiO_2_ has only been detected at temperatures above 1200 °C [26,27]. 

Furthermore, the enrichment of Si within the diffusion layer can lead to the precipitation of hard intermetallic Si-rich phases. Recent studies of intermetallic Cr-Ni-Si alloys indicated promising high temperature corrosion [28] and wear resistance [29,30] for alloys containing Cr_3_Si and Cr_13_Ni_5_Si_2_. That can make the coatings also interesting for applications that require high temperatures and erosion or wear, such as heat exchangers for particle heat receivers of concentrated solar power (CSP) plants, because previous studies in this specific environment have predicted a short, finite lifetime at 700 °C for several Ni-based alloys [31].

Within this work, Cr/Si-based slurry coatings have been applied on two Ni-based alloys Inconel 740H and Rene 80. The manufacturing process of the slurry coatings is described, and the resulting microstructures are characterized. For an initial evaluation of the oxidation behavior, the coatings were exposed for 1000 h at 900 °C in lab air and compared to the uncoated alloys.

## 2. Materials and Methods

The Cr-Si slurry coatings were applied on the two Ni-based superalloys Inconel 740H and Rene 80. The chemical compositions of the alloys in wt.-% are shown in Table 1. Inconel 740H is a wrought alloy, which is precipitation hardened by the Ni_3_Al gamma prime (γ’) phase and combines high temperature creep resistance with resistance to coal ash environments [32]. In contrast, Rene 80 is a cast γ’ precipitation hardened superalloy and is widely used for turbine blades. A major difference regarding the chemical compositions is the higher Cr content of IN740H, whereas Rene 80 has considerably higher amounts of Ti and Al and thus γ’-phase.

Samples were produced by wire electro-discharge machining (EDM). All samples were cut to a planar geometry of 20 × 10 × 2 mm with a 2 mm diameter drilled hole for hanging during exposure. Four samples of each material were coated. One was characterized in the as-coated condition. Prior to the coating application, the samples were sand blasted (Al_2_O_3_ 100 µm grit, 3 bar) and cleaned in an ultrasonic ethanol bath. Preliminary experiments showed that the use of pre-alloyed powder compositions allowed better control of the activity and reactivity and lead to more homogeneous and dense coatings compared to elemental powders. The targeted composition of 50Cr-30Si-20at.%Ni was arc-melted (MAM-1, Edmund Bühler) into ~7 g buttons. These buttons were then ball milled at 95 Hz for 15 min so particles of about ~50 µm diameter resulted. To produce the slurry, the powders were mixed in distilled water as the solvent with polyvinyl alcohol (PVA) as the binder in a weight ratio of 3:4 of PVA solution to powder. 

Each surface was sprayed with three layers using an air brush and dried in air after each layer. An average of about 0.15 g/cm^2^ amount of slurry was deposited in total. Following spraying, a two-step heat treatment in a reducing atmosphere of Ar-5vol.%H_2_ was conducted. Within the first step, the temperature was held at 350 °C for 3.5 h to burn out the binder to avoid trapped organic residuals and minimize resulting porosity within the coating. For the second step, the furnace was heated to 1160 °C (for Inconel 740H) or 1180 °C (for Rene 80) for the interdiffusion heat treatment and held for 4 h. The applied heat treatment temperature and dwell times were chosen according to the heat treatment temperatures of the respective alloys. By overlapping the slurry heat treatment with the alloy heat treatment parameters, the slurry coating should be easily incorporated into commercial production. After the heat treatment, the coated samples were ground to P1200 to reduce the roughness of the surface and to remove unattached powders. However, some residuals still remained attached to the surface. The uncoated samples were ground to P1200 as well.

The oxidation exposures were carried out in a muffle furnace in lab air at 900 °C for 1000 h based on the norm ISO 21608.2012 [33]. The test was started with two coated and uncoated samples of each material and interrupted after 700 h for the Rene 80 and 300 h for the Inconel 740H. Then, one of the two samples was taken out and a new sample put in before the test was continued for the remaining 300 or 700 h. The weight gained per surface area of the samples at the different points in time (300 h, 700 h and 1000 h) were determined and the corresponding cross-sections were produced using standard metallographically procedures. 

Characterization included X-ray diffraction (XRD, Bruker D8 XRD Advance with Cu-Kα radiation), scanning electron microscopy (SEM, Hitachi FlexSEM 1000), compositional profiles with a spatial resolution of about 1 µm by wavelength dispersive X-ray spectroscopy (WDX) and elemental distribution images were measured by electron probe microanalysis (EPMA, JEOL JXA-8100). The identification and assumption of the phases within the coatings was done based on the measured compositions within the WDS line scans and correlation to existing phases in the PDF2 database as well as by calculations using the JMatPro-v10.2 software.

## 3. Results and Discussion

### 3.1. Cr-Si Slurry Coatings

#### 3.1.1. Cr/Si-Coated Rene 80

**Figure 1:**
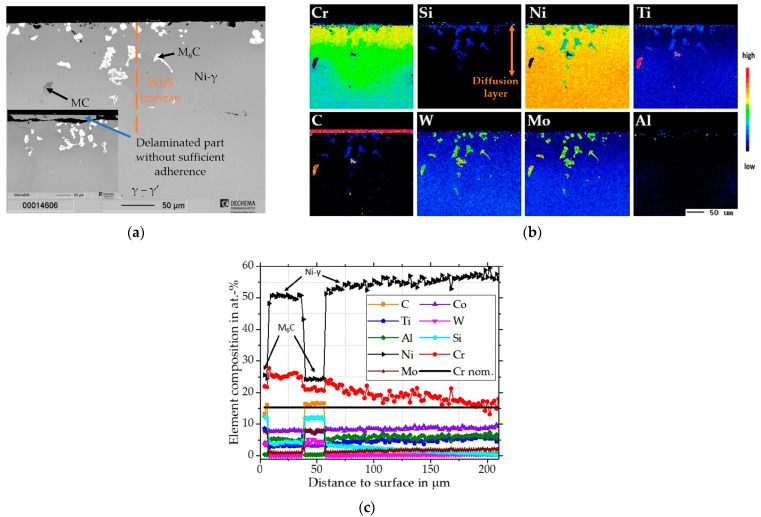
Characterization of Cr/Si-coated Rene 80. (**a**) SEM cross-section of as-coated Rene 80; (**b**) Element distribution maps of major alloying elements; (**c**) Concentration depth profiles of coated Rene 80 measured by WDS (adjusted for the C offset).

For the coating on the Rene 80 an exemplary cross-sectional BSE image with elemental distribution images and a corresponding concentration depth profile from the surface through the diffusion layer is shown in Figure 1. As marked in Figure 1b, a diffusion layer of about ~150 µm enriched in Cr and Si was estimated, after which the elemental compositions approached the nominal base composition of the Rene 80 (the nominal base composition of Cr of the Rene 80 in at.-% is indicated in Figure 1c by the black line). The coating consists of a γ-Ni matrix enriched in Cr and Si with precipitates of coarsened Si-rich M_6_C carbides and locally fine Ti-enriched MC carbides along the grain boundaries. The phases were identified and concluded from the measured compositions by the WDS profile (shown in Figure 1c, the position of the line scan performed is indicated by the dashed line). Silicon with refractory alloying elements (especially W and Mo) is reported to promote M_6_C precipitation along the grain boundaries and segregation within the formed, coarsened particles [34,35]. Some portions of the top layer had insufficient bonding to the surface (Figure 1a). Since elements from Rene 80 were also measured in these layers, it is likely that delamination occurred during the cooling from the heat treatment. The resulting diffusion layer was formed predominantly by inward diffusion of Cr, Si, and Ni from the slurry.

#### 3.1.2. Cr/Si-Coated Inconel 740H

**Figure 2:**
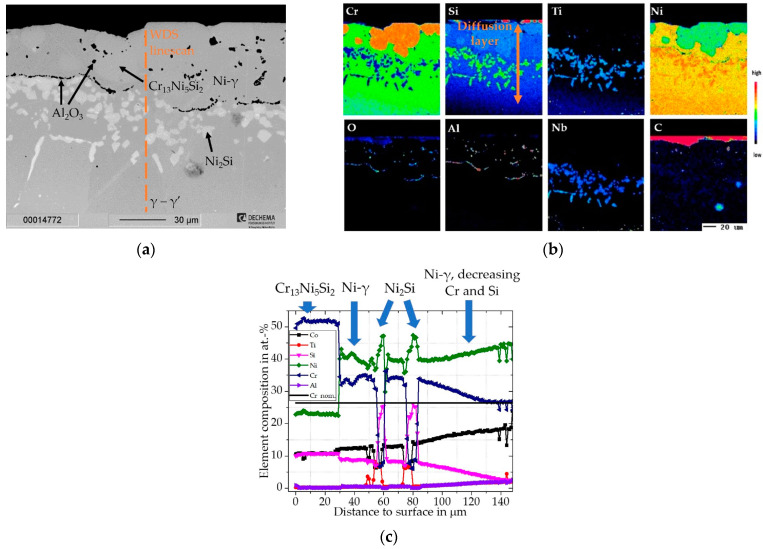
Characterization of Cr/Si-coated Inconel 740H (**a**) SEM cross-section of as-coated Rene 80; (**b**) Element distribution maps of major alloying elements; (**c**) Concentration depth profiles of coated Rene 80 measured by WDS.

For the coating on the Inconel 740H, an exemplary cross-sectional BSE image with elemental distribution images and a corresponding concentration depth profile from the surface through the diffusion layer is shown in Figure 2. As marked in Figure 2b, a diffusion layer of about ~150 µm enriched in Cr and Si was estimated, after which the elemental compositions approached the nominal base composition (the nominal base composition of Cr of the Inconel 740H in at.-% is indicated in Figure 2c by the black line). For Inconel 740H a diffusion layer of comparable thickness to the Rene 80 with ~150 µm enriched in Cr and Si was estimated as marked from the elemental distribution images in Figure 2c. The diffusion layer of the coating consists of precipitates of the intermetallic phase Cr_13_Ni_5_Si_3_ (σ) embedded in a Cr- and Si-enriched γ-Ni matrix. The phases were identified and concluded from the measured compositions by the WDS depth profiles (shown in Figure 2c, the position of the line scan performed is indicated by the dashed line). In comparison to the Rene 80, the nominal base composition of Inconel 740H with 24-wt.% Cr led to the formation of a high volume fraction (~30 vol.-% within the outer part of the diffusion layer) of the Cr-rich intermetallic phase Cr_13_Ni_5_Si_2_. In contrast, the considerably lower Cr content of Rene 80 (14 wt.-%) suppressed the formation of intermetallic phases, where Si segregates to the carbides rather than intermetallic silicides. The lower carbon and refractory element (Mo and W) contents prevent similar M_6_C precipitation for Inconel 740H. Even though a higher volume fraction of brittle, hard intermetallic phases increase the hardness of the diffusion layer for Inconel 740H, it is also more prone to crack initiation when compared to the ductile γ-Ni matrix with higher fracture toughness.

Along the former substrate/coating interface, inclusions of Al_2_O_3_ can be observed, which have accumulated to a dense film in some areas. This phenomenon has been also observed for Al-based slurry coatings and was attributed to the formation of Kirkendall porosity by Montero et al. [12], which subsequently healed and filled-in during the heat treatment. Three options exist for the contamination of oxygen at the surface to allow formation of Al_2_O_3_: Firstly, incomplete burnout would allow retention of residuals from the binder. Secondly, at the beginning of the heat treatment the Al activity is sufficiently high to react with oxygen impurities in the flowing gas mixture. Thirdly, the particles within the slurry all have thin native oxide scales from the production process. For the coating on the Rene 80, despite the higher Al-content, no such inclusions are present. This indicates a difference within the diffusion kinetics during the coating formation compared to the Inconel 740H and needs to be investigated further. 

Underneath the film of Al_2_O_3_, Ni_2_Si precipitates also formed within the enriched γ-Ni-matrix. Within a depth of about ~150 µm the measured composition approached the nominal composition of Inconel 740H.

Notably, when chromizing C-containing alloys by pack cementation, usually a thin Cr_23_C_6_ top layer has been observed [36,37]. However, with this novel slurry process, no Cr_23_C_6_ layer was detected. Harper et al. [23] noticed the absence of a Cr_23_C_6_ layer during co-deposition of Cr and Si by the pack cementation process, so it is not fully clear if this is an effect of the co-deposition or application process.

#### 3.1.3. Cr-Si Slurry Coating Process

The coating is formed through interdiffusion between the slurry particles and the substrate surface. Thus, the composition and properties of the resulting diffusion layer are influenced by the substrate in addition to the composition of the slurry and the heat treatment parameters. This leads to the different compositions in the diffusion layers on the two substrates examined, even though the initial concentration of the slurry was the same. 

**Figure 3:**
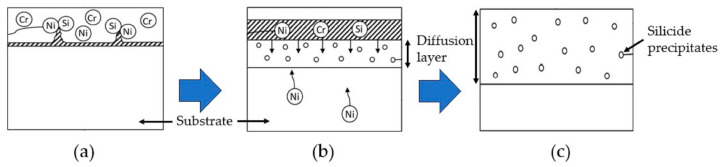
Diffusion process during interdiffusion heat treatment after [17]: (**a**) incubation period, where liquid phase forms along the interface; (**b**) growing diffusion layer; (**c**) idealized coating design after the heat treatment.

The assumed diffusion process during the heat treatment is schematically shown in Figure 3 and is described extensively in [17]. 

In situ, a liquid phase forms at the interface, mainly due to a low melting eutectic between Ni and Si. The addition of Ni to the slurry enables partial melting of the particles within the slurry. 

### 3.2. Oxidation Behavior: Quasi-Isothermal Oxidation Exposures

#### 3.2.1. Oxidation of Rene 80

##### Uncoated Rene 80

**Figure 4:**
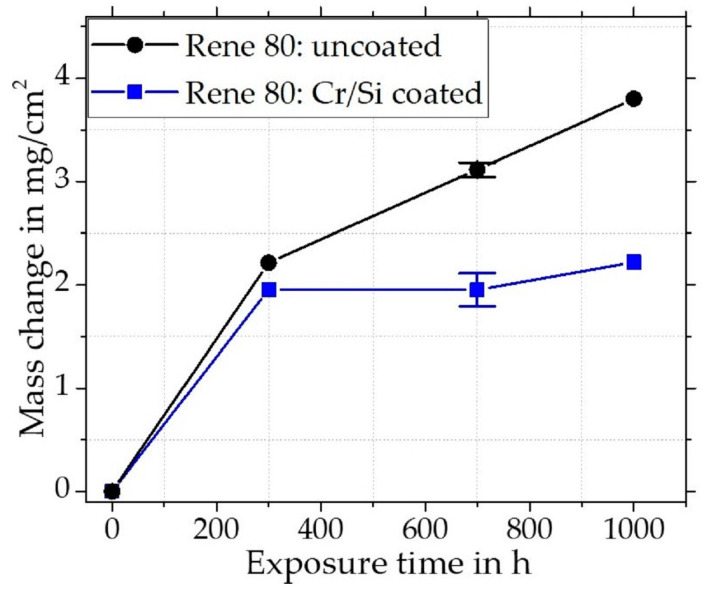
Net mass change of Cr/Si-coated and uncoated Rene 80 for up to 1000 h exposure in lab air at 900 °C.

**Figure 5:**
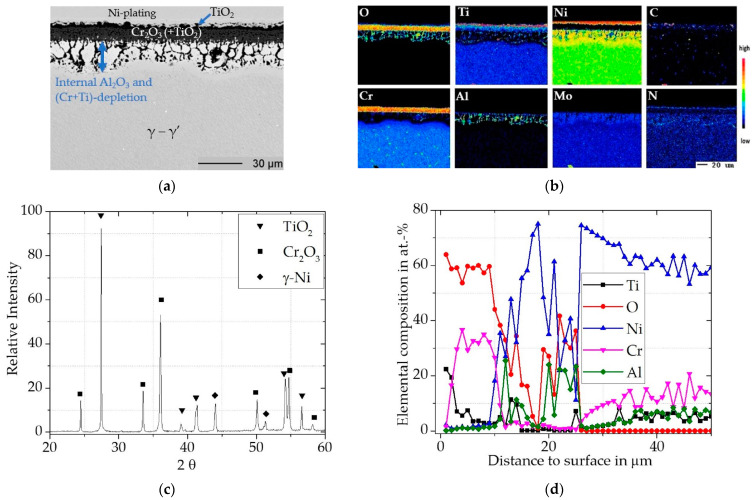
Characterization of uncoated Rene 80 for 300h at 900 °C: (**a**) BSE-image; (**b**) elemental distribution maps of major alloying elements; (**c**) XRD-Pattern; (**d**) concentration profile measured by WDS.

The uncoated Rene 80 showed a steep increase in mass within the first 300 h, after which the slope decreases somewhat, but the mass still continues to increase steadily (Figure 4). For Rene 80 after 300 h of exposure, a SEM cross-section is shown in Figure 5a with the corresponding element distribution maps (5b), a concentration depth profile, (Figure 5c) and a XRD pattern (Figure 5d). An outer thin TiO_2_ (Rutile, pdf2 file number used: 00-021-1276) layer on top of an ~10 µm thick Cr_2_O_3_ (Eskolaite, pdf2 file number used: 00-038-1479) scale is evident. Ti is reported to be able to diffuse through Cr_2_O_3_ at high temperatures [38,39]. Thus, the outer TiO_2_ scale probably formed simultaneously with Cr_2_O_3_ by outward diffusion of Ti after the initial transient stage. Within the Cr_2_O_3_ scale an incorporation of Ti up to about 12 at.-% was measured by WDS (Figure 5d) and additionally spots of incorporated TiO_2_ particles within the scale can be observed. As Ti substitutes Cr^3+^ by Ti^4+^, the incorporation of Ti functions as a p-type doping within the scale and generates Cr-vacancies [40,41]. Jalowicka et al. [42] observed a comparable Ti incorporation for Rene 80 during exposures at 1050 °C and suggested that the generated vacancies increased the diffusion rates through the scale. Due to the continuous outward diffusion of Cr and Ti to form the externally growing scales, these elements deplete in the subsurface zone. Subsequently, the Cr and Ti activity gets progressively reduced and Al starts to oxidize internally, which is also promoted by the increased oxygen partial pressure along the metal/oxide interface. Underneath the Cr_2_O_3_ scale, a zone of up to ~25 µm of internal oxidation (Al to Al_2_O_3_) formed, where the y’-precipitates dissolved and therefore the load bearing area of the material is lowered. Locally, TiN formed beneath the internal oxidation, which has developed into a denser layer in these locations. Alumina formation has locally reduced the oxygen partial pressure, so that the nitrides became more stable than oxides at these specific locations. 

In general, internal oxidation is considered to follow a parabolic mass gain [43]. However, for Rene 80 at 900 °C the constant increase in mass can be mainly attributed to the growing Cr_2_O_3_ scale and Al internal oxidation.

**Figure 6:**
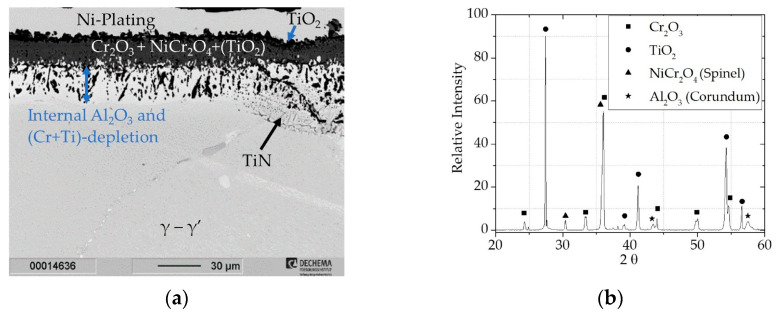
Characterization of uncoated Rene 80 after 1000 h of exposure at 900 °C in lab air: (**a**) BSE image; (**b**) XRD pattern.

The Cr_2_O_3_ scale thickness measured on the cross sections in Figure 5a and Figure 6a showed growth from ~10 µm (after 300 h) to ~16 µm (after 1000 h). Using XRD (Figure 6b), the formation of NiCr_2_O_4_ (Spinel, pdf2 file number used: 00-075-0198) within the oxide scale could be determined after 1000 h of exposure. By this point the Cr, Al, and Ti depletion within the γ-phase underneath the scale has progressed further, leading to the increased activity of Ni and the formation of nonprotective oxides. If scale spallation occurs, for example in thermocyclic conditions, Ni would be further oxidized leading to breakaway oxidation and fast degradation of the material. Due to the internal growing zone of Al_2_O_3_ it also became evident in the XRD after 1000 h of exposure (Corundum, pdf2 file number used:00-046-1212). 

##### Cr/Si-Coated Rene 80

**Figure 7:**
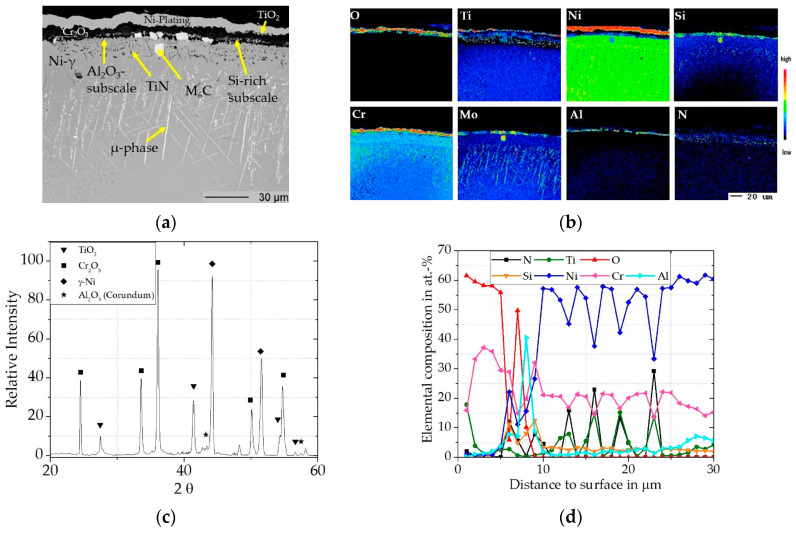
Characterization of Cr/Si-coated Rene 80 after 300 h at 900 °C: (**a**) BSE image; (**b**) elemental distribution maps of major alloying elements; (**c**) XRD pattern; (**d**) concentration profiles measured by WDS.

**Figure 8:**
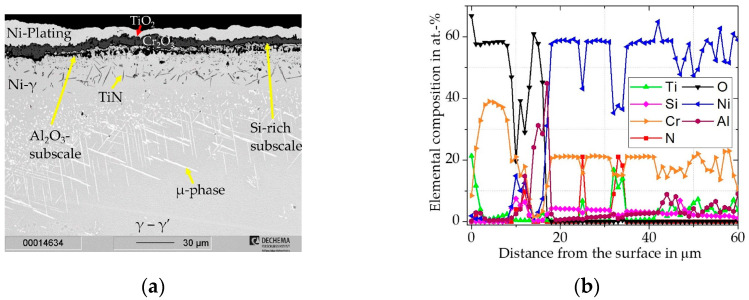
Characterization of Cr/Si-coated Rene 80 for 1000 h @ 900 °C: (**a**) BSE-image; (**b**) concentration profile measured by WDS.

The mass gain (Figure 4) for the coated Rene 80 plateaus after the first 300 h over the exposure time up to 1000 h. It should be noted that some un-diffused Cr/Si particles can remain on the surface after the slurry heat treatment, despite grinding and during oxidation, have a large effect on the initial mass increase due to the large surface area of the particles. Thus, the initial mass gain of the coated Rene 80 is higher as it reflects the oxidation of the coated surface and some remaining Cr/Si particles. In Figure 7a, a BSE image of the coated Rene 80 after exposure for 300 h is shown with the associated element distribution maps (Figure 7b), XRD pattern (Figure 7c), and concentration depth profile (Figure 7d) In contrast to the uncoated material, the Cr_2_O_3_ (Eskolaite, pdf2 file number used: 00-038-1479) scale of the coated Rene 80 showed significantly slower growth kinetics. The thicknesses were ~5 µm after 300 h and only ~6 µm after 1000 h (shown in Figure 8a). The full oxide scale is multi-layered with a thin outer TiO_2_ (Rutile, pdf2 file number used: 00-021-1276) layer on top of the Cr_2_O_3_ scale and then below the Cr_2_O_3_ (Eskolaite, pdf2 file number used: 00-038-1479) scale, a Si-rich subscale (evident in distribution maps, but not in the XRD), and then a dense Al_2_O_3_ (Corundum, pdf2 file number used:00-046-1212) subscale are distinctly observable. 

Compared to the uncoated Rene 80, the initially formed Cr_2_O_3_ scale showed minimal incorporation of Ti (see concentration profile in Figure 7d) and thus likely had a lower defect structure as a result. This can be attributed to the SiO_2_, which should represent an additional diffusion barrier according to [25]. After the exposures within this work at 900 °C in air no SiO_2_ phase is present in the XRD pattern, besides the enrichment seen in the EPMA maps. Douglas et al. [26] conducted oxidation exposures at 1000°–1200° on Ni-20Cr-3Si alloys and observed a Si-rich subscale underneath the outer Cr_2_O_3_ scale. Via X-ray diffraction, they were able to identify SiO_2_ for samples tested at 1200 °C and concluded that an amorphous SiO_2_ layer formed during exposure at temperatures below 1200 °C and crystallized SiO_2_ just formed at 1200 °C. Lowell et al. [27] made similar observations, in line with our results. 

With this extra diffusion barrier, the oxygen partial pressure at the oxide/metal interface is also reduced, and thus also the oxygen inward diffusion into the material is slower, which allows the formation of a dense Al_2_O_3_ subscale rather than internal oxidation. Additionally, the presence of chromium in the subscale decreases the required Al-activity for the formation of an Al_2_O_3_-scale instead of internal oxidation [44]. 

According to Wagner’s well-known theory [45,46], the formation of a protective scale occurs when the flux of the outward diffusing element (Cr, Si, Al) is higher than the inward diffusion of oxygen. This Wagnerian criterion appears to be met for the Cr-Si coating of the Rene 80 under these conditions. Below the multi-layer oxide scale, TiN precipitates are observable. Cr_2_O_3_ scales are described to be permeable to nitrogen at 900 °C [47]. In contrast, Al_2_O_3_ scales are reported as effective diffusion barriers for nitrogen [47,48]. Therefore, the TiN precipitates are most likely formed during the initial, transient stage of oxidation, when the Al_2_O_3_ subscale has not fully developed yet, and nitrogen could diffuse into the material. Jawolicka et al. [42] and Cruchley et al. [49] also reported TiN precipitation during oxidation exposures of Al_2_O_3_-forming Ni-based alloys and attributed it to the early stages of oxidation. After the Al_2_O_3_ scale has formed, the growth rates of the whole scale are slowed down significantly, and further oxidation behavior is dominated by the Al_2_O_3_ scale because the metal ions would need to diffuse through it [43].

Within the Cr/Si diffusion zone, the precipitation of µ-phase (bright spots, assumed according to the elemental distribution maps in Figure 7b) can be seen. This topological close-packed phase (TCP) precipitates from with Cr and Si enrichment metastable γ-phase during the exposure. This needle-shaped TCP precipitation within the grains during long term thermal exposure is well-known for refractory-rich (e.g., W and Mo) alloys [50]. 

Large scale precipitates can lead to degradation of the mechanical properties due to depletion of strengthening elements within the matrix, formation of brittle phases, and initiation of easy crack nucleation sites. But the influence of the evolution in the observed needle shape during thermal exposure is reported to be insignificant on the properties [50].

After the multi-layer oxide scale forms, the coated Rene 80 seemed to follow steady state behavior. The microstructure of an exposed sample after 1000 h in Figure 8a and the corresponding concentration profile in Figure 8b do not differ significantly from the sample exposed for 300 h. No further nitridation or oxidation could be measured. Also, the µ-phase precipitates did not grow beyond the coating diffusion zone into the base metal. The Cr-reservoir underneath the oxide after an exposure time of 1000 h still measured above 20 at.%. Thus, in the case of scale spallation the Cr-activity can be expected to be sufficient to provide fast self-healing of the scale.

#### 3.2.2. Oxidation of Inconel 740H

##### Uncoated Inconel 740H 

**Figure 9:**
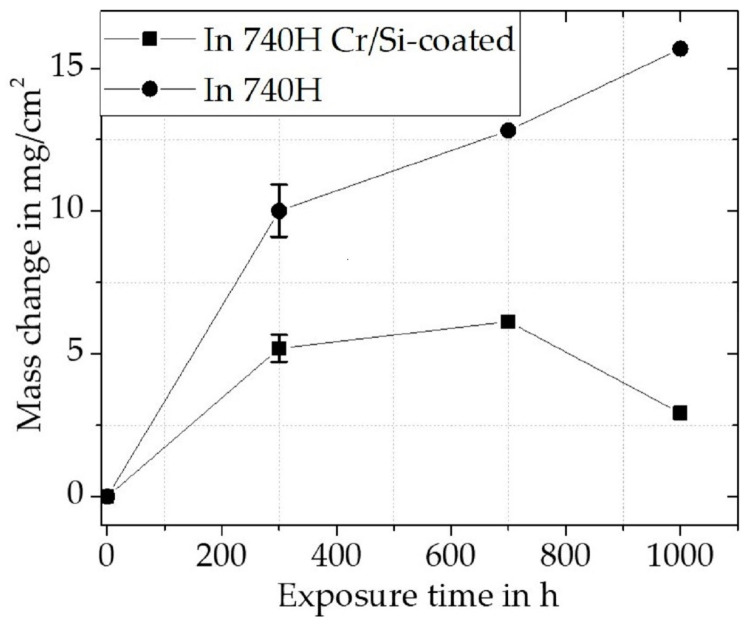
Net mass change during quasi-isothermal oxidation exposure of Cr/Si-coated and uncoated Inconel 740H for up to 1000 h at 900 °C in air.

**Figure 10:**
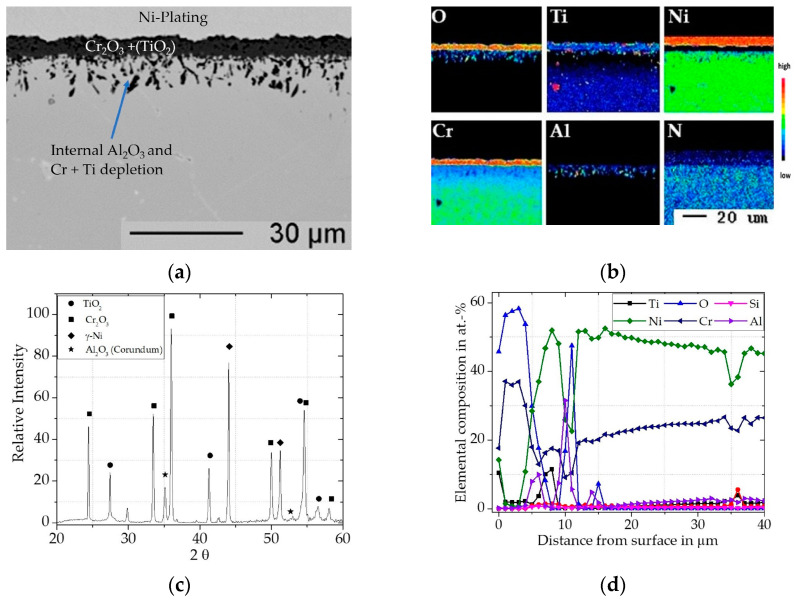
Characterization of uncoated Inconel 740H for 300 h @ 900 °C: (**a**) BSE-image; (**b**) elemental distribution maps of major alloying elements; (**c**) XRD-Pattern; (**d**) concentration profile measured by WDS.

For the uncoated Inconel 740H, a relatively large mass increase was measured for the first 300 h (Figure 9). The slope of the mass gain then decreases with further exposure time, while it does not plateau. This indicates that the inward diffusion of oxygen does not slow down significantly after the initial continuous oxide scale is established. From the cross-sectional BSE image (Figure 10a) and the XRD pattern (Figure 10c), it is clear that Inconel 740H formed a thin outer TiO_2_ (Rutile, pdf2 file number used: 00-021-1276) layer above a thicker Cr_2_O_3_ (Eskolaite, pdf2 file number used: 00-038-1479) scale. Within the concentration profile (Figure 10d) an incorporation of Ti (ca. 2 at.%) can be seen in the Cr_2_O_3_ scale.

Beneath the Cr_2_O_3_ layer, a ~10 µm deep zone of internal oxidation consisting of mainly Al_2_O_3_ and small amounts of TiO_2_ is evident. Comparable observations for the oxidation of the Inconel 740H were made by Gore et al. [51].

**Figure 11:**
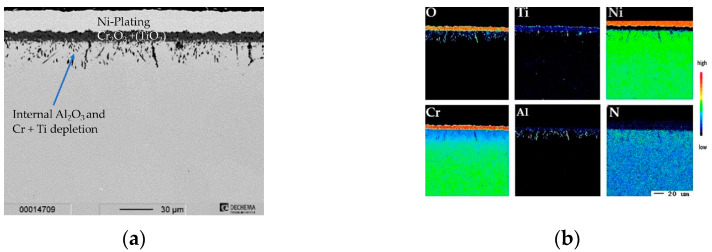
Characterization of uncoated Inconel 740H for 1000 h @ 900 °C: (**a**) BSE-image; (**b**) elemental distribution maps of major alloying elements Cr/Si-coated Inconel 740H.

The cross-section and elemental distribution maps after 1000 h (Figure 11) do not reveal major differences compared to the sample exposed for 300 h. The internal oxidation zone of Al_2_O_3_ and Cr-depletion grew to about ~20 µm after 1000 h of exposure.

##### Cr/Si-Coated Inconel 740H

In contrast to the uncoated Inconel 740H, the mass gain over the exposure time for the coated material in Figure 9 is significantly lower compared to the uncoated material. The mass loss between 700 h and 1000 h was due to outer scale spallation, most likely from the quasi-isothermal exposure causing thermal stresses, without subsequent additional oxidation attack. 

**Figure 12:**
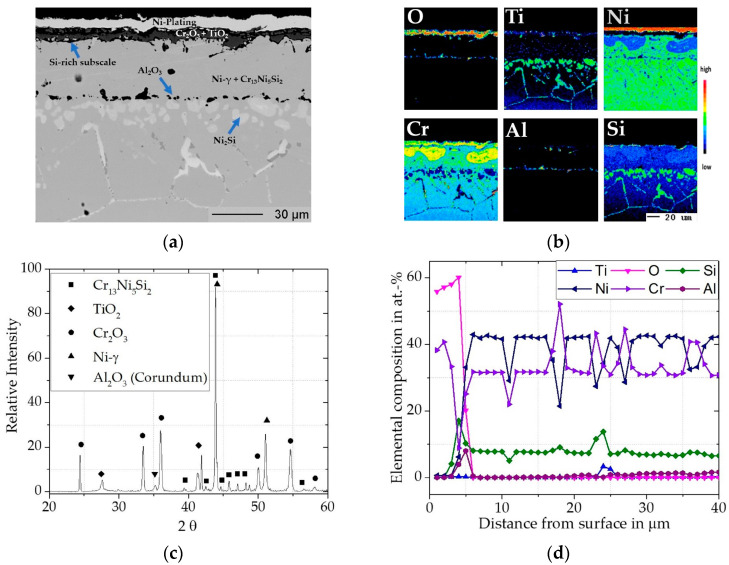
Characterization of Cr/Si-coated Inconel 740H for 300 h @ 900 °C in air: (**a**) BSE image; (**b**) elemental distribution maps of major alloying elements; (**c**) XRD pattern; (**d**) concentration profile as measured by WDS.

The Cr/Si-coating on Inconel 740H showed only local precipitation of TiO_2_ within the Cr_2_O_3_ (Eskolaite, pdf2 file number used: 00-038-1479) scale, rather than the more continuous layer that formed on bare Inconel 740H. Accordingly, the TiO_2_ (Rutile, pdf2 file number used: 00-021-1276) peak in the XRD pattern (Figure 12c) is less pronounced than for the uncoated samples. Underneath the chromia scale, an inner SiO_2_-rich subscale can be seen in the EPMA map (Figure 12b) and the concentration profile in Figure 12d gave a peak Si-concentration of 18 at.%. 

**Figure 13:**
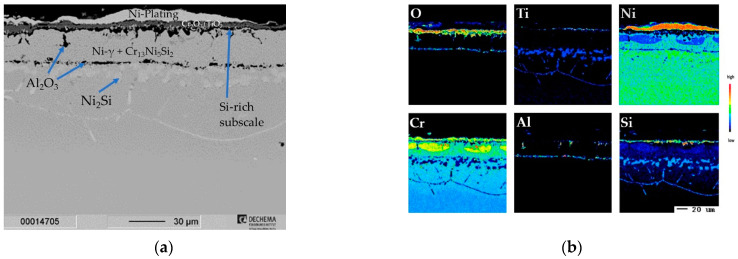
Characterization of Cr/Si-coated Inconel 740H for 1000 h @ 900 °C in air: (**a**) BSE image; (**b**) elemental distribution maps of major alloying elements.

Within the Cr_2_O_3_ scale, no significant Ti-incorporation could be measured, which leads to analogous conclusions as for the coating on Rene 80, where the higher Cr activity combined with the SiO_2_-rich subscale have ensured fewer defects in the Cr_2_O_3_, and thus also lowered the oxygen inward diffusion. The Cr/Si-coating did not show internal oxidation, rather the small amount of Al_2_O_3_ (that is present formed during the coating heat treatment (Figure 3) and also evident in Figure 12d (Corundum, pdf2 file number used: 00-046-1212). The Al_2_O_3_ inclusions along the former interface of the coating did not change over the exposure time and do not appear to have a major impact on the behavior under the investigated conditions (compare cross sections in Figure 12a and 13a).

Over the exposure time of 1000 h, the SiO_2_-rich subscale became more pronounced in the EPMA map (Figure 13b) when compared to the exposure time of 300 h. This matches the slow grow kinetics already observed by Harper et al. [24]. The scale structure of outer Cr_2_O_3_, internal SiO_2_-rich subscale and the remaining large Cr reservoir beneath after 1000 h of exposure can be expected to maintain protection for long exposure times. In contrast to the coated Rene 80, no Al_2_O_3_ subscale was formed underneath. This is likely due to the increased Al-content (3 wt.-%) as compared to 1.35 wt.-% in Inconel 740H. However, the SiO_2_-subscale on the Inconel 740H appears more pronounced than on the Rene 80 especially in its growth over time. This difference is most likely related to the Al_2_O_3_ subscale on the Rene 80, which limits the outward diffusion of Si and therefore the further growth of the subscale. In the case of the coating on the Inconel 740H, it can be assumed that the SiO_2_-rich subscale becomes the dominating layer for further oxidation behavior.

Over the exposure time, no additional Kirkendall porosity was observed during the exposure, so the interdiffusion rates between the coating and the substrate seem to be limited by the phases and oxides at the interface and no delamination of the coating layer should be expected at even longer exposure times. If further degradation of the coating did occur, the Al_2_O_3_ inclusions could even be beneficial as an additional diffusion barrier for oxygen and nitrogen.

## 4. Conclusions

A new process to apply Cr/Si-diffusion coatings using a slurry technique was demonstrated on the Ni-base alloys Inconel 740H and Rene 80. Diffusion layers enriched with Cr and Si for about ~150 µm were achieved. This new process avoids the use of halogenides and other environmentally harmful substances. Due to the simple and local application of the coating, the process has high potential to substitute the laborious pack cementation process, which is the current state-of-the-art process. The oxidation behavior of the coatings was evaluated in quasi-isothermal exposures in lab air at 900 °C. The increased Cr activity within the coating layer enabled a slow-growing Cr_2_O_3_ scale with low Ti content (defect concentration) and reduced the diffusion rates through the scale significantly.

Additionally, due to the Si content a slow-growing SiO_2_-rich film formed underneath the Cr_2_O_3_ scale. This subscale represents an additional diffusion barrier and supplies further protection, especially against the inward diffusion of O and N.

Lastly, this reduced oxygen partial pressure along the oxide/metal interface can avoid the internal oxidation of alloying elements with high oxygen affinities, e.g., Ti and Al. Instead, if these elements are alloyed in a sufficient amount, an additional subscale can be achieved, which was observed for Rene 80 in the form of an Al_2_O_3_ layer. After the formation of a subscale, the subsequent oxidation behavior is dominated primarily by the diffusion through the slower growing alumina or silica scale.

## 5. Patents

The presented Cr/Si slurry coating within this work represents a novel approach to apply Cr-based diffusion coatings on Ni-base superalloys. Therefore, a patent has been applied for the coating process “Verfahren zur Diffusionsbeschichtung mit einem Cr-Si-haltigen Schlicker” [17] in Germany under the application number 10 2022 112 093.7, 12 and internationally as a PCT application with the number PCT/DE2023/100353. 

## Figures and Tables

**Table 1:** Nominal chemical compositions in wt.-% of Rene 80 and Inconel 740H.

Alloying Elements	Rene 80	Inconel 740H
Cr	14	24.5
Co	9.5	20
Al	3	1.35
Ti	5	1.35
Nb	-	1.5
Mo	4	0.1
Si	-	0.15
W	4	-
C	0.17	0.03
Hf	0.03	-
B	0.02	-
Zr	0.03	-

## Data Availability

Date are contained within the article.
